# Metagenomic next-generation sequencing of alveolar lavage fluid improves the detection of pulmonary infection

**DOI:** 10.1515/biol-2025-1074

**Published:** 2025-05-12

**Authors:** Ziyu Meng, Dong Li, Wei Yang, Jihong Tang

**Affiliations:** Department of Respiratory Medicine, Putuo Hospital, Shanghai University of Traditional Chinese Medicine, 200062, Shanghai, China; Colortech (Suzhou) Biotechnology, 200062, Jiangsu, China; Department of Respiratory Medicine, Putuo Hospital, Shanghai University of Traditional Chinese Medicine, No.164 Lanxi Road, Putuo District, 200062, Shanghai, China

**Keywords:** metagenomic next-generation sequencing, pulmonary infection, pathogen detection, diagnostic methods

## Abstract

This study evaluated the effectiveness of metagenomic next-generation sequencing (mNGS) in detecting pathogens in patients with pulmonary infections, comparing a low-data-volume, human-depleted quantitative (Q) method and a high-data-volume, non-human-depleted pathogen capture engine (PACE) method. A total of 133 patients were enrolled, comprising 59 in a control group (traditional culture) and 74 in an mNGS group (51 Q and 23 PACE). Bronchoalveolar lavage fluid samples were collected for pathogen detection. *Mycobacterium tuberculosis* was predominantly detected via general mNGS, whereas *Candida albicans* and Epstein-Barr virus were more frequently identified by PACE and Q, respectively. Among participants, 22.97% had bacterial mono-infections, and 2.70% had viral mono-infections; the most common co-infection involved bacteria and viruses (25.68%). Patients with fever, abnormal white blood cell, neutrophil percentage, and D-dimer levels exhibited higher detection rates. PACE showed consistently high sensitivity (decreasing from 100 to 92% as thresholds became more stringent) and specificity and accuracy that peaked at 100 and 96%, respectively. The Q method maintained 100% sensitivity at the lowest threshold but showed variable specificity (0.52–0.67) and accuracy (71–75%). These findings highlight the need for caution in clinical applications when using low-data-volume, human-depleted approaches, especially for complex pulmonary infection cases.

## 1 Introduction

Pulmonary infections pose a significant challenge to global health, marked by high rates of morbidity and mortality, particularly in older adults and those with compromised immune systems [1,2,3,4]. These infections present with various symptoms, such as cough, fever, and respiratory distress, requiring timely and precise diagnosis for effective management [5]. Traditional diagnostic techniques, though commonly employed, are constrained by their sensitivity and prolonged processing times, which can impede the timely initiation of necessary treatments [6,7].

The etiological agents responsible for pulmonary infections are varied, including bacteria, viruses, and fungi, each posing distinct diagnostic and therapeutic challenges [8,9]. Bacterial pneumonia, a primary cause of morbidity and mortality, requires prompt and accurate identification to facilitate effective treatment [10]. Conventional detection methods, such as culture techniques and polymerase chain reaction (PCR) panel analysis, are limited by their range of detectable pathogens and sensitivity, especially concerning rare or emergent pathogens [11].

The introduction of metagenomic next-generation sequencing (mNGS) represents a transformative shift in the diagnostic landscape of infectious diseases, offering numerous advantages over traditional methods [12,13]. mNGS provides an unbiased, exhaustive approach that detects a broad spectrum of infectious agents without the need for prior culturing, thereby overcoming major limitations associated with traditional diagnostic techniques for identifying hard-to-culture microbes [14]. In diagnosing pulmonary infections, mNGS has demonstrated superior sensitivity in detecting pathogenic bacteria and fungi compared to conventional culture methods [15,16]. Although its sensitivity for viral infections may be lower than that of PCR, mNGS compensates by identifying a wider range of viral species, highlighting its diagnostic versatility [17]. Research indicates that mNGS can deliver more diagnostic information, shorten hospital stays, and achieve higher positivity rates than culture methods, particularly in complex cases such as pulmonary infections with pleural effusion [18,19]. Additionally, mNGS enhances the sensitivity and specificity of bacterial identification in critically ill patients, including organ transplant recipients, proving highly effective in detecting viruses and diagnosing mixed infections [20,21]. This technology surpasses conventional microbiological tests in both diagnostic positivity and pathogen detection rates [22,23]. The deployment of mNGS in the diagnosis of pulmonary infections marks a significant advance in clinical microbiology, providing a rapid, accurate, and comprehensive diagnostic tool that facilitates targeted therapeutic interventions.

In recent years, the development of human-depleted mNGS techniques has been recognized for potentially enhancing pathogen detection by minimizing background noise from human DNA, thus improving the specificity and sensitivity of microbial identification. Clinical studies have underscored the utility of these human-depleted mNGS methods in various diagnostic scenarios. For example, a study demonstrated that using 0.025% saponin for specimen preprocessing significantly reduces human DNA content, enhancing the sensitivity of pathogen detection in mNGS analyses [24]. Another research effort revealed that mNGS equipped with a host depletion filter achieved the highest pathogen identification rate of 74.4% compared to mNGS without such a filter and traditional blood culture methods in septic patients [25]. Additionally, the use of the MolYsis kit combined with saponin and Turbo DNase for pre-treatment has been shown to decrease the proportion of host DNA and enrich microbiome reads in samples, thereby improving both DNA and RNA pathogen detection via mNGS in patients with pneumonia [26]. More recently, a rapid and unbiased mNGS workflow incorporating a human DNA depletion kit and a library preparation kit has been developed, enabling the enrichment and detection of bacteria and fungi in plasma through low-depth sequencing [27]. These advancements in human-depleted mNGS methods mark a significant progression in the field of infectious disease diagnostics, providing a rapid, precise, and comprehensive tool for enhancing pathogen detection and informing targeted therapeutic approaches.

In this study, we aim to evaluate the effectiveness of the traditional culture approach and mNGS in detecting pathogens in patients with pulmonary infections. Additionally, we will compare two specific mNGS methodologies: the Q human-depletion technique and the pathogen capture engine to assess their respective efficacies in pathogen detection.

## 2 Materials and methods

### 2.1 Patients and sample collection

This research involved a comparative analysis of mNGS techniques for diagnosing pulmonary infections. We assessed 133 patients from the Department of Pulmonary Medicine at Putuo Hospital, affiliated with Shanghai University of Traditional Chinese Medicine, over the period from January 2021 to December 2022. The study participants were divided into a control group, comprising 59 patients, and an mNGS group, consisting of 74 patients. The mNGS group was further subdivided into two cohorts: the Q subgroup with 51 patients and the pathogen capture engine (PACE) subgroup with 23 patients. Each participant was diagnosed with a pulmonary infection, confirmed through clinical evaluations and imaging studies.

#### 2.1.1 Inclusion criteria

The inclusion criteria are as follows: (1) age ≥18 years; (2) adult patients who were eligible for bronchoalveolar lavage fluid (BALF) sampling; (3) clinical signs/symptoms; (4) suggestive of pulmonary infection, such as fever, cough, purulent sputum, or new/worsening respiratory symptoms; (5) radiological findings consistent with pulmonary infection (evidence of an infiltrate or consolidation on chest imaging); and (6) willingness to provide informed consent.


**Informed consent:** Informed consent has been obtained from all individuals included in this study.
**Ethical approval:** The research related to human use has been complied with all the relevant national regulations and institutional policies and in accordance with the tenets of the Helsinki Declaration and has been approved by the Ethical Committee of Putuo Hospital, affiliated with Shanghai University of Traditional Chinese Medicine.

#### 2.1.2 Exclusion criteria

The exclusion criteria are as follows: (1) inability to obtain BALF samples; (2) recent antibiotic use that could confound pathogen detection; (3) insufficient clinical data; (4) severe immunosuppression with infections or other conditions unrelated to pulmonary pathogens; (5) medications that could cause immunosuppression; and (6) refusal of consent.

BALF samples were collected for analysis: 10 mL was allocated for mNGS testing and 20 mL was used for traditional culture methods.

### 2.2 Culture of BALF

Initially, thorough skin disinfection was performed. Following this, BALF collection was carried out, strictly adhering to aseptic techniques to prevent contamination. Each patient contributed 20 mL of BALF, which was equally distributed between aerobic and anaerobic culture bottles for routine microbiological testing. The samples were immediately transported to the laboratory at ambient temperature to maintain viability.

In the laboratory, both aerobic and anaerobic cultures were incubated using the advanced BD Bactec™ FX system, following the manufacturer’s guidelines to optimize growth conditions. Positive cultures were subjected to subculturing to facilitate precise microbial identification through standard microbiological methods. Antibiotic susceptibility testing was conducted using the disc diffusion technique to determine the appropriate therapeutic interventions.

The entire culture process was conducted over a period of 5 days to ensure comprehensive microbial growth and accurate identification.

### 2.3 Q mNGS procedure for BALF samples

The human-depleted Q method involves a differential lysis technique previously described [28]. Initially, the collected specimens were treated with 0.025% saponin (Sigma) and Chaps cell extract buffer (10×) from New England BioLabs. This mixture was vortexed for 10 s and then incubated for 5 min at room temperature. Subsequently, 10× Turbo DNase buffer (Thermo Fisher Scientific, Inc.) was added to achieve a final concentration of 1×, along with 2 μL of Turbo DNase (Thermo Fisher Scientific, Inc.) to all tubes. The samples were then gently mixed and incubated at 37°C for 30 min.

DNA extraction was performed on 0.2 mL of each specimen using the QIAsymphony Virus/Bacteria Kit on the automated QIAsymphony SP platform (Qiagen). Following extraction, library preparation commenced, which involved DNA fragmentation, end-repair, A-tailing, adapter ligation, and PCR amplification, utilizing the NEBNext Ultra II DNA Library Prep Kit for Illumina. The quality and concentration of the resulting libraries were assessed using the Agilent 2100 Bioanalyzer and the Qubit 2.0 Fluorometer, respectively.

High-quality libraries were then sequenced on the Illumina NovaSeq platform, incorporating a negative control in each sequencing batch to validate the accuracy of the sequencing data. Finally, the samples were forwarded to an mNGS service provider, Matridx Biotechnology in Hangzhou, China, for sequencing analysis.

### 2.4 PACE mNGS procedure for BALF samples

For the PACEseq analysis, BALF samples were transported to HUGO BIOTECH in Beijing, China. The DNA extraction from these samples was conducted using the QIAamp DNA Micro Kit from QIAGEN, Germany. Following extraction, the DNA was utilized for library construction employing the QIAseq™ Ultralow Input Library Kit for Illumina, also from QIAGEN, Germany.

The quality of all constructed libraries was assessed using the Qubit fluorometer from Thermo Fisher, USA, and the Agilent 2100 Bioanalyzer from Agilent Technologies, USA. Only DNA libraries that met quality standards were advanced to the sequencing stage.

The qualified DNA libraries were then sequenced using the NextSeq 550 platform by Illumina, USA, ensuring high-resolution results for the identification and analysis of pathogens present in the BALF samples.

### 2.5 Post-sequencing analysis

After sequencing, initial processing involved the removal of low-quality, low-complexity sequences, adapters, and small fragment DNA sequences from the raw files. Human-derived sequences were then filtered out; these were identified by alignment to the human reference genome (hg38) using Bowtie2 software. The non-human sequences that remained were subsequently aligned to the microbial genome database available at NCBI. This alignment facilitated the classification of all microorganisms present in the samples down to the species level.

For quality control and validation purposes, each batch of experiments included negative controls, which consisted of sterile deionized water, and positive controls, which used synthesized DNA fragments of known quantities. Both control types underwent the same wet laboratory procedures and bioinformatics analyses as the clinical samples to ensure the integrity and accuracy of the results.

Normalization of read counts was performed to account for variations in genome size among different species, using the Reads Per Kilobase of the genome per Million mapped reads calculation method. This step was crucial for accurately determining the relative abundance of each detected microorganism in the samples based on these normalized read counts.

### 2.6 Results interpretation

The microbial data derived from the mNGS analysis of BALF samples were rigorously compared against in-house background databases and negative controls. This comparison was crucial for effectively excluding common contaminants that are typically encountered in clinical and laboratory settings. To refine the final list of detected pathogens, specific threshold criteria were established based on the precise alignment of sequence reads to each pathogen species.

The thresholds for detecting different types of pathogens were stringently set. For bacteria, mycoplasma, chlamydia, DNA viruses, and fungi, a minimum of three sequence reads was required to confirm the presence of the pathogen. For the *Mycobacterium tuberculosis* complex, even a single read was deemed sufficient due to its clinical significance and the need for rapid intervention.

The entire mNGS process, from the collection of BALF samples to the generation of results, was completed within a 24-h period. Senior clinicians, including associate chief physicians, conducted detailed reviews of the mNGS reports. Their evaluation considered several key factors, such as the number of unique reads, the relative abundance of detected microbes, and the genome size of each organism.

By integrating these mNGS data with clinical observations and other diagnostic results, clinicians were able to make conclusive diagnoses of the causative agents. This comprehensive approach not only ensured accurate interpretation of the mNGS findings but also facilitated precise and timely medical interventions.

### 2.7 Statistical analysis

The statistical analysis conducted in this study incorporated both descriptive and inferential approaches. For continuous variables that followed a normal distribution, means were used for presentation. In contrast, non-normally distributed variables were described using medians. Categorical variables were expressed in frequencies and percentages. Sensitivity, specificity, positive predictive value (PPV), and negative predictive value (NPV) were calculated using data organized in 2 × 2 contingency tables.

For comparisons, various statistical tests were employed, including Fisher’s exact test, the chi-squared test, and the Mann–Whitney *U*-test for comparisons. SPSS was used for data management, descriptive statistics, and inferential statistical tests (e.g., chi-squared tests, Mann–Whitney *U*-tests, Fisher’s exact tests), while GraphPad Prism 7 was used for generating certain figures and performing additional statistical analyses, primarily for visualization, correlation analyses, and *P*-value determinations. The level of statistical significance was set at *P*-values less than 0.05, and all tests were two-tailed. This approach ensured a comprehensive and robust statistical evaluation of the study’s findings.

## 3 Results

### 3.1 Baseline characteristics of patients

This study involved 133 patients diagnosed with pulmonary infections. These patients were allocated into two primary groups for analysis: a control group consisting of 59 patients and a mNGS group, which included 74 patients. The mNGS group was further divided based on the detection method employed: mNGS quantitative detection (51 patients) and mNGS PACE detection (23 patients).

Analysis of the baseline characteristics, as detailed in Table 1, demonstrated that the groups were comparable in terms of age, gender, and smoking history. The average ages in the mNGS subgroups were 58.14 years (±14.79) for the Q group and 61.96 years (±11.31) for the PACE group; however, this age difference was not statistically significant (*P* = 0.275). Regarding gender distribution, there was no significant difference between the control and mNGS groups (*P* = 0.955), as well as within the mNGS subgroups (*P* = 0.277). Similarly, smoking history did not show significant differences between the control and mNGS groups (*P* = 0.516) or among the mNGS subgroups (*P* = 0.820).

**Table 1:** Clinical characteristics of participants

Characteristics	Control (*n* = 59)	mNGS detection (*n* = 74)	*P*	mNGS Q detection (*n* = 51)	mNGS PACE detection (*n* = 23)	*P*
Age	69.19 ± 15.22	59.32 ± 13.84	0.00018	58.14 ± 14.79	61.96 ± 11.31	0.275
**Gender**			0.955			0.277
Male	34	43		27	16	
Female	25	31		24	7	
Smoke history (*n*)	11	17	0.516	11	6	0.82
**Primary diseases (** ***n***)						
Diabetes	8	10	0.994	6	4	0.49
Cardiovascular disease	15	19	0.974	10	9	0.091
Digestive system diseases	6	6	0.68	3	3	0.367
Renal system diseases	7	13	0.361	8	5	0.526
Cerebrovascular diseases	7	6	0.469	4	2	1
Respiratory diseases	4	5	0.996	3	2	0.643
Fever (*n*)	34	34	0.181	22	12	0.638
WBC, ×10^9^/L	7.49 ± 4.13	9.01 ± 5.40	0.068	8.49 ± 4.15	10.17 ± 7.46	0.219
Neutrophil, %	70.06 ± 11.87	71.14 ± 14.28	0.633	72.65 ± 11.78	67.81 ± 18.56	0.259
D-dimer (μg/L)	0.79 ± 0.64	1.20 ± 1.60	0.057	1.08 ± 1.41	1.19 ± 1.89	0.778
Hypoproteinemia (*n*)	16	19	0.805	14	5	0.816
Empyema (*n*)	1	2	0.697	1	1	1

These data indicate that the groups were well-matched in demographic and health-related behaviors, allowing for a focused comparison of the diagnostic methods employed.

### 3.2 Diagnostic performance of different mNGS methods

When assessing the diagnostic accuracy of mNGS using varying thresholds, the PACE method showed an initial sensitivity of 100% for the least stringent criteria, which involved detecting ≥3 specific sequence counts and having ≥1% relative abundance. This sensitivity slightly decreased to 92% under the most stringent conditions of ≥30 sequence counts and ≥1% relative abundance. The specificity of the PACE method reached 100% under these strict conditions (Table 2). When the criteria were set to a specific sequence count of ≥30 and a relative abundance of ≥10%, the accuracy of the PACE method reached 96% (Table 2), representing the highest accuracy achieved under the thresholds established in this study.

**Table 2:** Sensitivity and specificity, AUC, PPV, and NPV of mNGS PACE method

Items	Specific sequence count ≥3, relative abundance ≥1%	Specific sequence count ≥30, relative abundance ≥1%	Specific sequence count ≥3, relative abundance ≥10%	Specific sequence count ≥30, relative abundance ≥10%
Sensitivity	1.0	0.92	0.92	0.92
Specificity	0.7	0.80	0.90	1.00
Accuracy	0.87	0.87	0.91	0.96

AUC, area under the curve.

On the other hand, the Q method displayed a perfect sensitivity of 100% at a less stringent threshold of ≥20 specific sequence counts and ≥0.5% relative abundance. However, the specificity at this level was significantly lower at 52% (Table 3). When the threshold was increased to ≥200 specific sequence counts and ≥10% relative abundance, the accuracy of the Q method was 76% (Table 3), marking it as the highest accuracy obtained for this method under the study’s thresholds.

**Table 3:** Sensitivity and specificity, AUC, PPV, and NPV of mNGS Q method

Items	Sensitivity	Specificity	Accuracy
Specific sequence count ≥20, relative abundance ≥0.5%	1.00	0.52	0.75
Specific sequence count ≥20, relative abundance ≥5%	0.88	0.56	0.71
Specific sequence count ≥20, relative abundance ≥10%	0.83	0.67	0.75
Specific sequence count ≥200, relative abundance ≥0.5%	0.92	0.59	0.75
Specific sequence count ≥200, relative abundance ≥5%	0.88	0.59	0.73
Specific sequence count ≥200, relative abundance ≥10%	0.83	0.70	0.76

While the Q method showed perfect sensitivity at lower detection thresholds, its specificity varied significantly. In contrast, the PACE method consistently maintained high sensitivity and specificity across different testing conditions. Notably, the PACE method exhibited higher overall accuracy under various thresholds compared to the Q method.

### 3.3 Comparative distribution of pathogens detected by different mNGS methods

**Figure 1:**
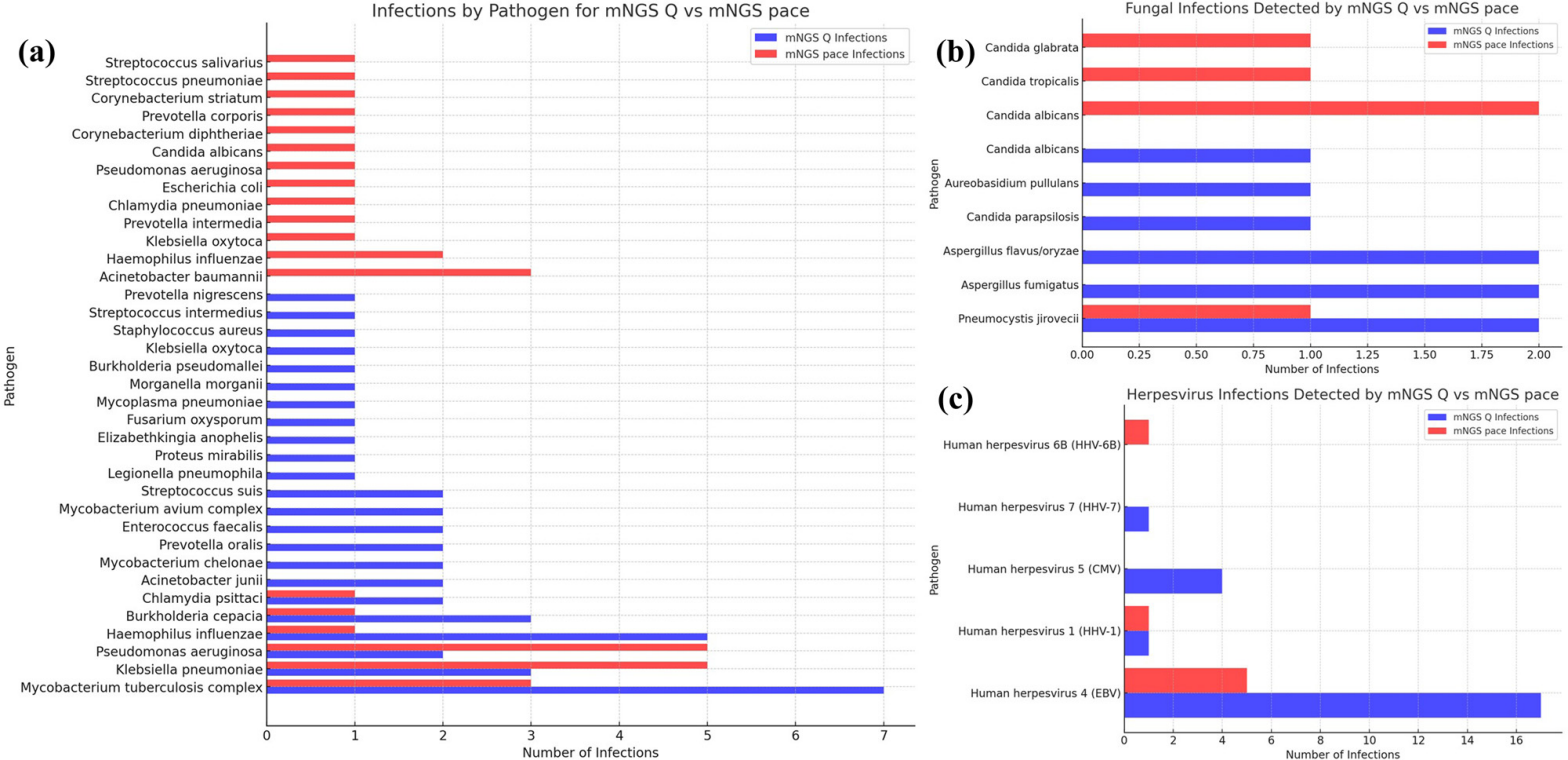
Genus distribution of bacteria (a), fungi (b), and virus (c) detected by different mNGS methods.

The analysis provided a comprehensive overview of the distribution of bacterial, fungal, and viral pathogens identified in pulmonary infection cases utilizing various mNGS methods, as illustrated in Figure 1. The results highlighted a wide spectrum of pathogens, with detection frequencies varying across the mNGS, mNGS quantitative, and mNGS PACE detection methods. Notably, the *M. tuberculosis* complex was the most prevalent bacterial pathogen, displaying a higher detection rate, particularly in the mNGS Q method. This suggests a strong sensitivity of this method towards bacterial pathogens.

In contrast, fungal pathogens such as *Candida albicans* were more frequently detected by the mNGS PACE method, pointing to its enhanced sensitivity for identifying fungal species. The viral detection landscape was primarily dominated by Epstein-Barr virus (EBV), which was significantly detected by both the mNGS Q and PACE methods, indicating their effectiveness in viral identification.

Other pathogens, including *Klebsiella pneumoniae*, *Pseudomonas aeruginosa*, and cytomegalovirus (CMV), also showed varying rates of detection, which emphasizes the differential diagnostic capabilities of each mNGS approach. This variability in pathogen detection underscores the importance of selecting the appropriate mNGS method based on the suspected infectious agent in clinical settings.

### 3.4 Comparative analysis of pathogen infection percentages in patients using NGS Pace and NGS Q methods

**Figure 2:**
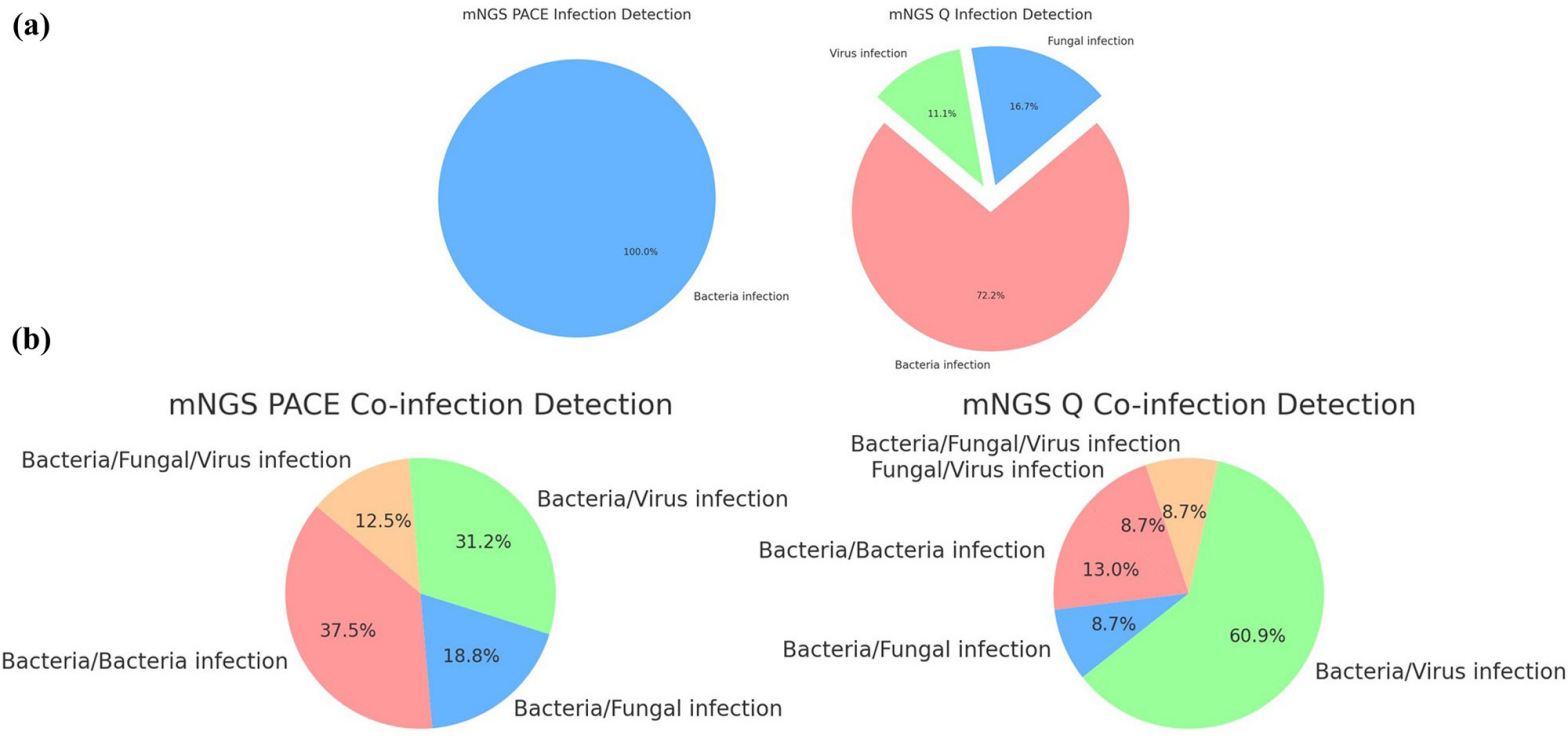
Percentage of patients with infections with different types of pathogens (a), or co-infections (b) detected by mNGS PACE and Q method.

This section of the study provides a detailed analysis of the distribution of pathogen infections among patients, comparing the detection capabilities of the NGS PACE and NGS Q methods. The findings, illustrated in Figure 2a, detail both the percentage and actual number of patients affected by different types of pathogens. Among the 74 patients in the mNGS group, a diverse range of bacterial, fungal, and viral infections was observed.

Bacterial infections were identified in 17 patients (22.97%), with the NGS Q method showing a higher detection rate (13 patients) compared to the NGS PACE method (4 patients). Fungal infections, less common in occurrence, were exclusively detected by the NGS Q method in 3 patients (4.05%). Viral infections were found in 2 patients (2.70%). Additionally, a subset of the mNGS group (17.57%) had unidentified pathogens, predominantly detected by the NGS Q method.

Regarding co-infections, the study identified dual bacterial infections in 9 cases (12.16%), bacterial and fungal co-infections in 5 cases (6.76%), and bacterial and viral co-infections as the most frequent, occurring in 19 cases (25.68%). Co-infections involving all three pathogen types – bacteria, fungi, and viruses – were present in four cases (5.41%). Furthermore, there were two instances (2.70%) of fungal and viral co-infections noted (Figure 2b).

These results underscore the variable effectiveness of the NGS PACE and NGS Q methods in detecting different pathogens and highlight the complexity of pathogen distribution in pulmonary infections.

### 3.5 Integrated analysis of pathogen detection in relation to clinical and laboratory parameters using NGS methods

In this comprehensive analysis of pathogen detection using NGS methods, we correlated detection rates with clinical parameters like fever, hypoproteinemia, and empyema, along with key laboratory findings such as white blood cell (WBC) count, neutrophil percentage (NEUT%), and D-dimer levels.

**Figure 3:**
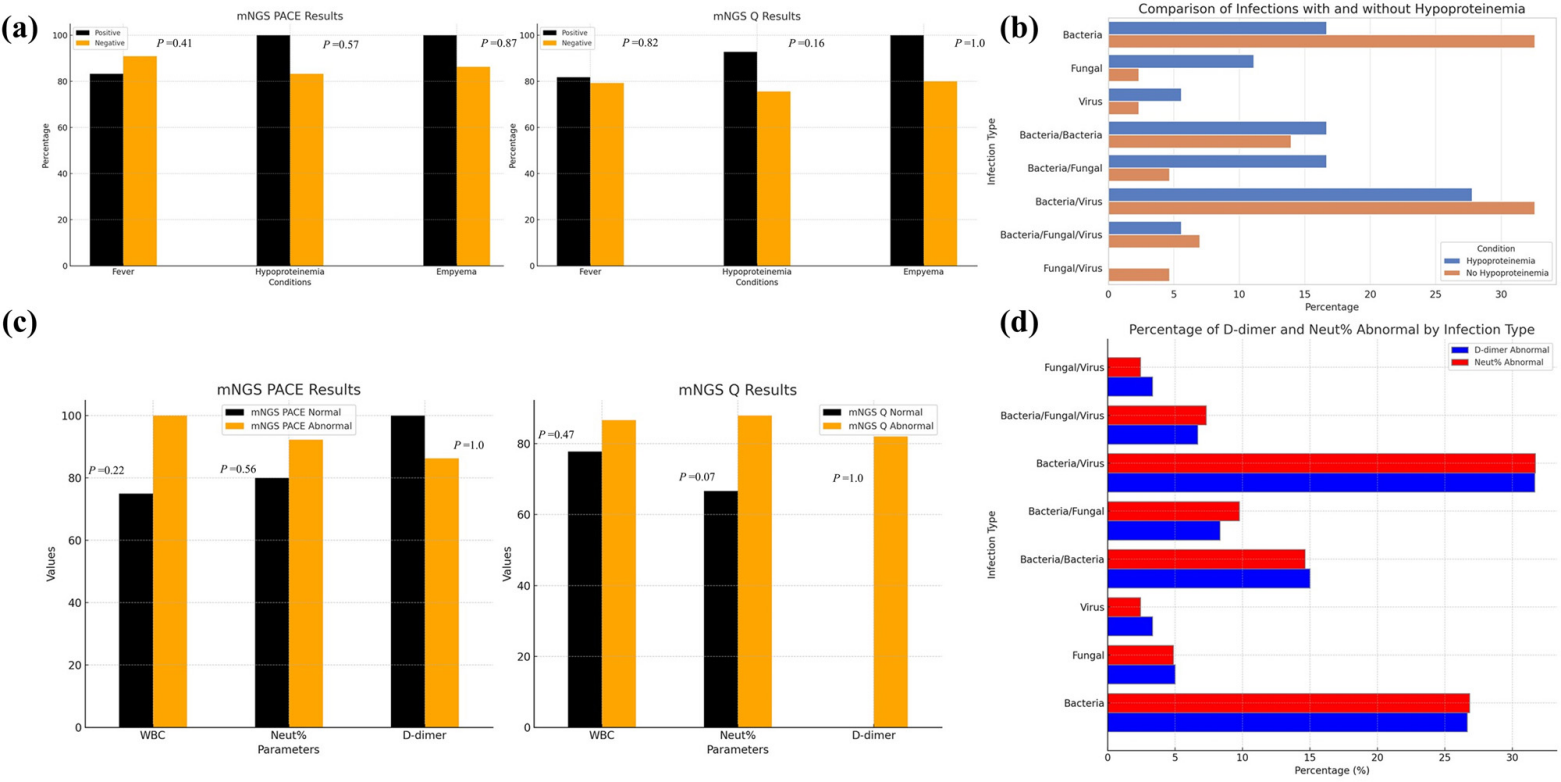
(a) The influence of fever, hypoproteinemia, and empyema on the detection rate of mNGS in pulmonary infections. Positive/negative represent cases with/without these symptoms, respectively. Chi-squared test. (b) Proportion of infection type of pulmonary infection with hypoproteinemia detected using mNGS PACE and Q methods. (c) The influence of WBC, NEUT%, and D-dimer on the detection rate of mNGS in pulmonary infections. Chi-squared Test. (d) Proportion of infection type of pulmonary infection with abnormal level of NEUT% and D-dimer detected using mNGS. WBC, white blood cell; NEUT, neutrophil. *P < 0.05.

For clinical parameters, NGS displayed variable detection rates: in patients with fever, pathogens were identified in 28 out of 34 cases (82.3%), while in patients without fever, mNGS identified pathogens in 33 out of 40 cases (82.5%), as shown in Figure 3a. Analyzing the performance of specific mNGS methods, the PACE method detected pathogens in 10 out of 12 cases with fever (83.3%), and 10 out of 11 cases without fever (90.9%). In contrast, the Q method identified pathogens in 18 out of 22 cases with fever (81.8%) and 23 out of 29 cases without fever (79.3%). These findings indicate that both the Q and PACE methods perform comparably in detecting pathogens in patients with fever, but the PACE method has a higher detection rate in patients without fever.

In this detailed analysis, we evaluated the efficacy of NGS methods in detecting pathogens among patients with and without hypoproteinemia. NGS demonstrated a higher efficacy in identifying pathogens in patients with hypoproteinemia, detecting pathogens in 18 out of 19 cases (94.7%), while showing lower efficacy in those without hypoproteinemia, where pathogens were identified in 43 out of 55 cases (78.2%), as shown in Figure 3a. Specifically, the PACE method achieved a detection rate of 100% in patients with hypoproteinemia and 83.3% in those without. Conversely, the Q method identified pathogens in 92.9% of hypoproteinemic patients and 75.7% in non-hypoproteinemic patients.

Further analysis, depicted in Figure 3b, highlights pathogen detection variability based on hypoproteinemia. Among the 19 patients with hypoproteinemia, pathogens were detected as follows: bacterial infections in 3 individuals, fungal infections in 2, viral infections in 1, and no pathogens detected in 1 case. Co-infections included combinations of bacterial with bacterial (three cases), bacterial with fungal (three cases), and bacterial with viral (five cases), plus one case of co-infection involving all three types. In the 55 patients without hypoproteinemia, bacterial infections were detected in 14, fungal in 1, and viral in 1, with 12 cases having no pathogens detected. Co-infections in this group included bacterial with bacterial (6 cases), bacterial with fungal (2 cases), bacterial with viral (14 cases), bacterial with both fungal and viral (3 cases), and fungal with viral (2 cases).

The study also examined the impact of elevated NEUT% and D-dimer levels on pathogen detection rates. Patients with abnormal WBC counts showed a pathogen detection rate of 92.31% compared to 77.08% in those with normal counts, as indicated in Figure 3c. The PACE subgroup detected pathogens in 75% of cases with normal WBC and 100% with abnormal WBC, while the Q subgroup showed a detection rate of 77.78% with normal WBC and 86.67% with abnormal WBC.

Regarding neutrophil levels, pathogens were detected in 71.43% of patients with normal levels and 89.13% with elevated levels. The PACE subgroup exhibited detection rates of 80% with normal and 92.31% with elevated neutrophil levels. The Q subgroup detected pathogens in 66.67% of cases with normal levels and 87.88% with elevated levels.

Additionally, over 80% of patients had abnormal D-dimer levels, with pathogen detection rates 86.4% in the PACE group and 82% in the Q group, as shown in Figure 3c.

The distribution of different types of infections detected in patients with abnormal neutrophil levels and D-dimer values indicated that bacterial and bacterial/virus co-infections were most common, as detailed in Figure 3d. This comprehensive analysis underscores the complex interplay between clinical and laboratory parameters and their impact on the efficacy of NGS methods in pathogen detection.

### 3.6 Correlation of NGS detection with WBC, neutrophil, and D-dimer levels

**Figure 4:**
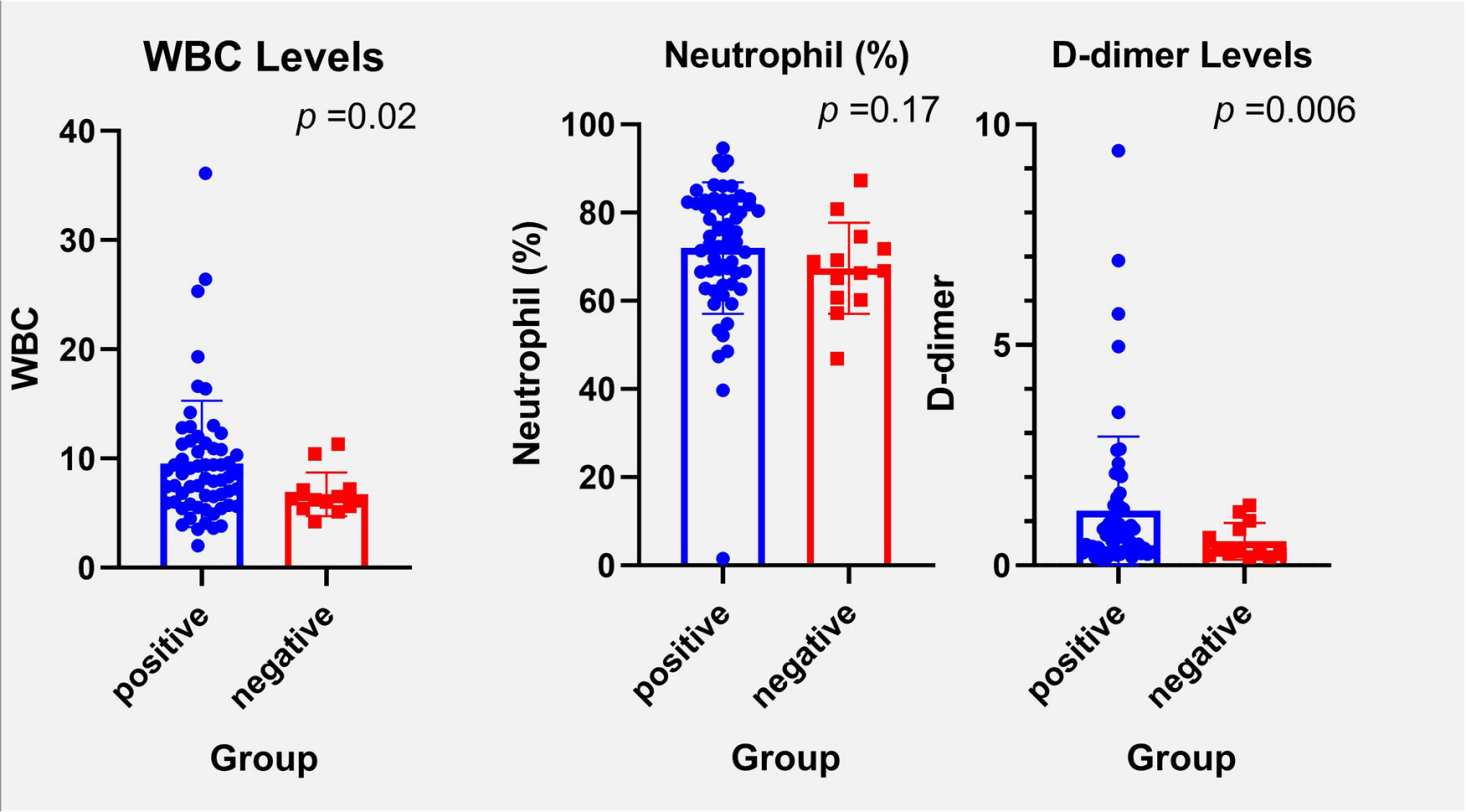
The comparisons of WBC, neutrophil, and D-dimer across mNGS groups. *P < 0.05.

In this segment of the study, we explored the correlation between NGS pathogen detection and key inflammatory markers: WBC count, NEUT%, and D-dimer levels. Patients were categorized into a positive group, where pathogens were detected (62 cases), and a negative group, where no pathogens were found (12 cases). The analysis indicated that the levels of WBC, NEUT%, and D-dimer were significantly higher in the positive group compared to the negative group, with statistical significance (*P* < 0.05), as illustrated in Figure 4.

Due to the limited number of cases in the negative group, a detailed comparative analysis of WBC, NEUT%, and D-dimer levels between the Q and PACE subgroups was not conducted. This suggests that elevated levels of these markers may be indicative of an active infection, which corresponds with the successful detection of pathogens using NGS technology. The significant differences in these markers between the groups underscore their potential utility as indicators of infection severity and diagnostic outcomes in clinical settings.

## 4 Discussion

In this study, we evaluated the effectiveness of mNGS applied to BALF for diagnosing pulmonary infections – a field where traditional pathogen detection methods often struggle with sensitivity and specificity. Our results show that mNGS successfully identifies a wide range of pathogens. There was also a notable correlation between the presence of pathogens and clinical parameters such as fever and hypoproteinemia, along with laboratory findings like WBC, neutrophil, and D-dimer levels. The diagnostic performance of mNGS varied depending on the detection thresholds used. The PACE method demonstrated high sensitivity, while the Q method provided a balanced trade-off between sensitivity and specificity. This improved detection capability enables more precise and effective treatment strategies, which could potentially enhance patient outcomes.

mNGS has emerged as a highly effective diagnostic tool for identifying a wide range of pathogens across various medical conditions. It has shown particular promise in detecting pathogens in immunocompromised patients, where it surpasses conventional microbiological tests in terms of detection rates [29]. Its application in analyzing BALF has demonstrated high sensitivity and specificity [18]. Beyond respiratory infections, mNGS has also been pivotal in diagnosing osteoarticular infections in pediatric patients [30]. In critical care settings, mNGS has been beneficial in identifying a broader spectrum of pathogens in bloodstream infections [31]. Its superiority extends to the detection of a higher number of bacteria and fungi, as well as in diagnosing mixed infections more effectively than traditional cultures [32]. Moreover, mNGS provides quick and precise diagnostics [33]. Additionally, it is capable of identifying difficult-to-cultivate pathogens, especially viruses [34]. However, mNGS faces significant limitations in pathogen detection, primarily due to the overwhelming presence of background human DNA, which can obscure pathogen signals and reduce sensitivity and specificity. This challenge is particularly pronounced in clinical specimens where the pathogen-to-human DNA ratio is low [28]. To address this, two approaches have emerged: Q methods and PACE techniques. Q methods, such as those utilizing saponin for human DNA depletion, have been shown to significantly enhance the sensitivity of pathogen detection by improving the microbial-to-human DNA ratio [28]. Conversely, PACE techniques, particularly capture-based targeted NGS, have demonstrated superior sensitivity (up to 99.43%) and specificity (83.87%) in identifying pathogens, making them preferable in scenarios requiring precise pathogen identification, such as lower respiratory tract infections [35]. Ultimately, the choice between these approaches depends on the clinical context, with PACE being advantageous for high sensitivity needs, while Q methods may be suitable for broader pathogen detection in mixed samples [36,37].

In this study, a variety of pathogens were detected in pulmonary infections. Notably, *M. tuberculosis* emerged as the most frequently identified bacterial pathogen. Fungal pathogens, such as *C. albicans*, were more commonly detected using the mNGS PACE method. The viral landscape was prominently marked by the presence of EBV. Other pathogens, such as *Klebsiella pneumoniae*, *Pseudomonas aeruginosa*, and CMV, also demonstrated varied detection rates. mNGS has proven to be a highly effective method for detecting mixed infections, surpassing traditional diagnostic approaches. The study documented mixed infections involving various combinations of bacterial, fungal, and viral pathogens. These findings highlight the complex and multifaceted nature of mixed pulmonary infections, emphasizing the need for advanced diagnostic methods like mNGS. This technology not only enhances the detection and understanding of such infections but also significantly aids in their management.

In our study, we critically evaluated the efficacy of two distinct mNGS approaches, the Q and PACE methods, for diagnosing pulmonary infections. The PACE method, known for its thorough sequencing depth and comprehensive data analysis, enables the detection of a broad spectrum of pathogens. This method exhibited high sensitivity, starting at 100% for the least stringent criteria (specific sequence counts of ≥3 and relative abundance of ≥1%), which slightly decreased to 92% under more stringent conditions (specific sequence counts of ≥30 and relative abundance of ≥1%), achieving a maximum specificity of 100%. The accuracy also improved with stricter thresholds, reaching up to 96%, making it suitable for complex clinical scenarios where accurate identification of diverse pathogens is crucial. Its ability to handle large data volumes without human DNA depletion allows for comprehensive detection of mixed infections. Conversely, the Q method emphasizes rapid processing and is tailored for the swift identification of specific pathogen types. It showed consistently high sensitivity of 100% at lower thresholds but variable specificity, ranging from 52 to 67%. Specificity improved under more stringent conditions but remained lower compared to the PACE method. The Q method includes human DNA depletion, enhancing sensitivity especially useful for quickly identifying pathogens in samples with low microbial loads. However, this method’s lower specificity at less stringent conditions could result in higher false positive rates, potentially complicating clinical interpretation in diverse infections. Both mNGS methods have their respective strengths and weaknesses, and the choice between them should be based on specific clinical requirements and the nature of the infectious disease being investigated.

Our study, while insightful, encounters several limitations that must be acknowledged. First, there is a lack of specific comparative literature, which constrains our ability to robustly position our findings within the broader scientific context. Additionally, the generalizability of our results across different types of pulmonary infections and diverse patient populations is limited. These factors underscore the burgeoning potential of mNGS in clinical diagnostics and highlight the pressing need for further research to refine these methods and fully explore their capabilities across various clinical scenarios. Furthermore, it is important to note that the sample size in our study was relatively small, a constraint largely imposed by the logistical challenges during the COVID-19 pandemic. This limitation may impact the generalizability of our results. Moving forward, we plan to expand our cohort in subsequent studies to enhance the statistical significance of our findings. A larger sample size would allow for a more robust comparative analysis of the mNGS methods under study. This expansion is crucial for advancing our understanding of mNGS applications and optimizing their use in healthcare settings.

Ultimately, the decision on which mNGS method to use should be guided by the clinical context, weighing factors such as the necessity for rapid diagnostic results against the complexity of the infection being investigated. This tailored approach will help maximize the diagnostic utility of mNGS technologies in clinical microbiology.
